# Osteoporosis knowledge and beliefs in diabetic patients: a cross sectional study from Palestine

**DOI:** 10.1186/s12891-018-1961-6

**Published:** 2018-02-07

**Authors:** Ghaith A. Ishtaya, Yazan M. Anabtawi, Sa’ed H. Zyoud, Waleed M. Sweileh

**Affiliations:** 1 Division of Human Medicine, College of Medicine and Health Sciences, An-Najah National University, Nablus, 44839 Palestine; 2 Department of Clinical and Community Pharmacy, College of Medicine and Health Sciences, An-Najah National University, Nablus, 44839 Palestine; 3 Department of Physiology, Pharmacology and Toxicology, College of Medicine and Health Sciences, An-Najah National University, Nablus, 44839 Palestine

**Keywords:** Osteoporosis, Diabetes mellitus, Knowledge, Beliefs, Palestine

## Abstract

**Background:**

Osteoporosis is a potential metabolic complication of diabetes mellitus (DM). Therefore, patients with DM should have adequate osteoporosis knowledge and beliefs in order to get engaged in osteoporosis preventive behaviors. The objective of this study was to assess osteoporosis knowledge and beliefs among diabetic patients.

**Methods:**

This was a cross sectional study carried out at Al-Makhfiah governmental primary healthcare unit in Nablus, Palestine from September 2016 to December 2016. The tools used to assess knowledge and beliefs were Osteoporosis Health Belief Scale (OHBS) and the Osteoporosis Knowledge Test (OKT) respectively.

**Results:**

Three hundred diabetic patients were interviewed regarding their knowledge and belief about osteoporosis. The study sample included 192 (64.0%) females. Mean ± standard deviation (SD) of the participants was 58.5 ± 9.3 years. Regarding co-morbidities, 229 (76.3%) had at least one co-morbidity other than DM. The majority of participants incorrectly answered 19 out of 32 questions of OKT scale. The mean OKT score was 13.5 ± 4.2 indicating poor osteoporosis – related knowledge. Females had significantly higher nutrition (*p* = 0.037), exercise (*p* = 0.043), and OKT score (*p* = 0.021) than males. Regarding OHBS, female participants had significantly higher belief score of susceptibility (*p* <  0.01) and seriousness (*p* <  0.01) of osteoporosis compared to males.

**Conclusions:**

Diabetic patients had poor osteoporosis knowledge and moderate perception of susceptibility and seriousness of osteoporosis. These results require implementation of awareness programs among DM patients to increase their practices regarding preventive measures of osteoporosis such as calcium intake and exercise.

**Electronic supplementary material:**

The online version of this article (10.1186/s12891-018-1961-6) contains supplementary material, which is available to authorized users.

## Background

Diabetes mellitus (DM) is a chronic medical illness that requires careful and continuous adherence to and implementation of daily treatment regimens, dietary restrictions, and change in life style that can be demanding and difficult to implement by some diabetic patients [1]. According to World Health Organization’s (WHO) recent report, the number of people diagnosed with DM has risen from 108 million in 1980 to 422 million in 2014 [2]. Prevalence of DM is increasing in all world regions including the Arab Middle Eastern region [3].

Long term microvascular and macrovscular complications of DM are well known and include nephropathy, retinopathy, neuropathy, acute coronary syndrome, and stroke [4]. Another serious, but an overlooked, complication of DM is bone diseases or decreased skeletal integrity and strength [5–8]. Diabetic osteopathy affects both types of diabetes although type II DM is often associated with normal or high bone mineral density (BMD) [9, 10]. A recent meta-analysis study demonstrated that individuals with type I DM have 5.76 relative-risk of hip fracture compared with non-DM individuals while people with type II DM have 1.34 relative-risk of hip fractures compared with non-DM people [11]. Postmenopausal women with type I DM have 12.25 times higher risk of osteopenia and osteoporosis than non-diabetic women [12, 13]. Glycemic control plays an important role in risk of bone fracture in diabetic patients [14, 15]. The Rotterdam Study demonstrated that individuals with inadequate glycemic control had 47–62% higher fracture risk than individuals without DM, Individuals with adequate glycemic control had a risk similar to those without diabetes (hazard ratio: 0.91 [0.67–1.23]) [14].

The exact pathogenesis of diabetes-induced risk of osteoporosis is still controversial. For patients with type I DM, the absolute deficiency of insulin and the potential presence of autoimmune diseases can lead to bone fragility and poor bone health [16–20]. In type II DM patients, reduced blood flow to bone may contribute to bone loss and fragility [16, 18–22]. Individuals with DM might also have impaired bone repair leading to accumulation of microcracks and increased cortical porosity [14, 23]. Recent studies demonstrated that circulating biochemical markers of bone formation and resorption such as P1NP, osteocalcin, bone-specific alkaline phosphatase, and bone resorption marker serum CTx have been found to be decreased in type II DM independent of BMD [24].

Worldwide literature in osteoporosis knowledge focused mainly on knowledge and belief in females of different age categories [25–28]. Osteoporosis knowledge studies had been carried out in various diseases including cancer, thalassemia, and HIV patients [29–31]. Searching Scopus for publications in osteoporosis knowledge in diabetic patients retrieved one article from Malaysia in which the authors assessed the psychometric properties of osteoporosis knowledge tool in type II diabetic patients [32]. Diabetic patients are considered a high risk category for osteoporosis and therefore knowledge of diabetic patients about osteoporosis is crucial for their general health [33]. Therefore, the aim of this study was to provide data about knowledge and belief about osteoporosis among diabetic patients in Palestine using internationally validated scales [34–36]. Our ultimate goal is to enhance osteoporosis awareness and preventive behavior among diabetic patients in Palestine. However, to achieve this ultimate goal, it is initially important to investigate and understand the baseline knowledge and beliefs of diabetic patients in Palestine toward osteoporosis. Our results will guide the design of future programs and educational materials promoting behaviors that can ultimately slow down, prevent osteoporotic complications, and therefore improve the quality of life of diabetic patients in Palestine.

## Methods

### Study sample and setting

This was a cross sectional study. Participants in this study were diabetic patients attending Al-Makhfiah primary healthcare unit in Nablus, north of Palestine. The study was carried out for four months, from September to December 2016. Al-Makhfiah clinic offers primary healthcare services and medications for individuals with chronic diseases who have governmental medical insurance. The number of diabetic individuals who attend Al-Makhfiah clinic on regular basis is less than 700. Recruited participants for this study were given full information regarding the purpose of the study and were asked to give informed consent before the interview. Participants were asked to complete a questionnaire through a face-to-face interview. The participants were interviewed by two co-authors who are senior medical students (G.I and Y.A). Participants in this study were limited to diabetic patients regardless of type. Participants who reported being diagnosed with osteoporosis or had previous bone fracture or currently taking calcium/vitamin D were excluded.

### Sample size calculation

To estimate the sample size, a margin of error less of than 5% and a confidence level of 95% were entered in Raosoft calculator [37]. The total number of target population was entered as 700 while the he response rate was entered as 70%. A sample size of at least 222 participants was needed to perform the study. Assuming a response rate of 50% will yield a sample size of 249.

### Measuring tools

Tools used to collect the required data were the “Osteoporosis Health Belief Scale” (OHBS) and the “Osteoporosis Knowledge Test (OKT)”. Both were obtained from the developer with permission to use in Palestine [38]. The OKT and OHBS had been translated into different languages and were found reliable and valid in different communities [32, 39–42]. The Arabic version of OKT and OHBS had been previously published and found to be reliable and acceptable [43]. We used the Arabic version of published OKT and OHBS after introduction of certain minor language changes to suite the Arabic accent of Palestinian culture. Permission to use the original scales and scoring methodology were obtained from the developer [44]. The translated scales are attached (Additional file 1).

The OKT is a 32-multiple choice instrument and has two subscales, a nutrition, and an exercise - related subscale. The two subscales had 14 items in common. In scoring the OKT, correct answers were coded as 1 while incorrect answers were coded as 0. Total score for OKT scale ranges from 0 to 32. The range for nutrition subscale was from 0 to 26 while that for exercise subscale ranges from 0 to 20. For each interviewed patient, scores of the OKT and the two subscales were analyzed as continuous variables and no categorization of scores was used. Examples of questions in the nutrition and exercise subscales include: “best source of calcium?: apple, cheese, cucumber”, “best way to reduce osteoporosis: swim, walk, stretching?”.

The OHBS consists of 42 items divided into seven subscales: susceptibility (items 1–6), seriousness (items 7–12), benefits of exercise (items 13–18), benefits of calcium intake (items 19–24), barriers of exercise (items 25–30), barriers of calcium intake (items 31–36), and health motivation (items 37–42). The internal reliability testing for the OHBS yielded a Cronbach’s alpha value of 0.720. The internal reliability of the seven OHBS subscales were 0.83, 0.71, 0.73, 0.72, 0.68, 0.69, and 0.66 for susceptibility, seriousness, benefits of exercise, benefits of calcium intake, barriers of exercise, barriers of calcium intake and health motivation subscales respectively. The OHBS is scored by awarding 5 for responses of “Strongly Agree” to 1 for “Strongly Disagree” for each item. Since there are 6 items in each subscale, the possible score for each subscale ranges from 6 to 30 with higher scores indicating higher belief in the tested subscale. The total score for the whole OHBS scale ranges from 42 to 210. Total score for each OHBS subscale was analyzed separately and as a continuous variable with no categorization of scores. The Arabic version of the OHBS subscales is shown in the additional file.

Examples of questions in the OHBS subscales include: “chances of getting osteoporosis high”; “when think about osteoporosis, get depressed”; “regular exercise helps build strong bones”; “taking enough calcium cuts chance of broken bones”; “have no place where can exercise”; “to eat more calcium foods have to give up other I like”; “look for new information related to health”.

### Ethical consideration

To maintain the rights of participants in the study an Institutional Review Board (IRB) application was submitted to An-Najah National University. The IRB approved the study and asked for verbal consent from the participants and not a written consent because the study does not involve any invasive procedure. Furthermore, the Palestinian Ministry of Health approved the study and gave permission to authors to conduct the study after obtaining verbal approval from the participants. Before the collection of data, the participants were handed an informed consent that included information about all aspects of the study. Developers of the questionnaire gave permission to use the tool for the study.

### Statistical analysis

Descriptive statistics including mean ± standard deviation (SD) or frequency (percentage) were presented for each variable, stratified by gender. Scores for all subscales were tested for normality using Kolmogrov - Smirnov test and were found to be not normally distributed. In Bivariate analysis, Mann-Whitney U test was used for ordinal variables while correlation between total knowledge scores and various belief subscales were carried out using Spearman correlation.

## Results

### Demographic and clinical characteristics of the participants

Three hundred diabetic patients were recruited; 192 (64%) were females. The mean ± SD age of the participants was 58.5 ± 9.3 years. The majority of participants were living in urban areas (215; 71.7%). The majority also reported being currently married (251; 83.7%). For the level of education, the majority (236; 78.7%) did not have a college degree. Regarding occupation, the majority (217; 72.3%) of participants were unemployed. Participants had mean ± SD duration of DM of 11.3 ± 8.0 years. More than half of the participants (174; 58%) were insulin users and two thirds of the participants were hypertensive. Regarding co-morbidities, 229 (76.3%) of studied patients have at least one co-morbidity other than DM and 91 patients (30.3%) had retinopathy.

Comparison of the demographic and clinical characteristics between female and male participants (Table 1) showed that females were significantly younger than males (mean ± SD age for females was 57.1 ± 8.9 while that for males was 61 ± 9.6 year; *p* <  0.01). Furthermore, females had significantly shorter duration of diabetes than males (mean ± SD duration of DM in females was 10.6 ± 7.6 while that for males was 12.6 ± 8.6 years; *p* = 0.036). Significant differences between males and females were also shown in place of residence (*p* = 0.022), marital status (*p* <  0.01), level of education (*p* <  0.01), employment status (*p* <  0.01), use of insulin (*p* = 0.012), and presence of hypertension (*p* <  0.01).

**Table 1:** Demographic and clinical characteristics of the study sample examined by gender

Variable	Total	Male	Female	P*
Age (M ± SD)	58.5 ± 9.3 (*n* = 300)	61 ± 9.6 (*n* = 108)	57.1 ± 8.9 (*n* = 192)	**< 0.01**
Residency				
Urban	71.7% (*n* = 215)	63% (*n* = 68)	76.6% (*n* = 147)	**0.022**
Rural	28.3% (*n* = 82)	37% (*n* = 40)	23.4% (*n* = 45)	
Employed				
Yes	27.7% (*n* = 83)	62% (*n* = 67)	8.3% (*n* = 16)	**< 0.01**
No	72.3% (*n* = 217)	38% (*n* = 41)	91.7% (*n* = 176)	
Currently married				
Yes	83.7% (*n* = 251)	95.4% (*n* = 103)	77.1% (*n* = 148)	**< 0.01**
No	16.3% (*n* = 49)	4.6% (*n* = 5)	22.9% (*n* = 44)	
Education				
≥ college	(21.3%) (*n* = 64)	31.5% (*n* = 34)	15.6% (*n* = 30)	**< 0.01**
< college	(78.7%) (*n* = 236)	68.5% (*n* = 74)	84.4% (*n* = 162)	
Duration of DM (year)	11.3 ± 8.0 (*n* = 300)	12.6 ± 8.6 (*n* = 108)	10.6 ± 7.6 (*n* = 192)	**0.036**
Using insulin				
Yes	58% (*n* = 174)	67.6% (*n* = 73)	52.6% (*n* = 101)	**0.012**
No	42% (*n* = 126)	32.4% (*n* = 35)	47.4% (*n* = 91)	
Hypertension				
Yes	66.0% (*n* = 19)	55.6% (*n* = 60)	71.9% (*n* = 138)	**< 0.01**
No	34.0% (*n* = 102)	44.4% (*n* = 48)	28.1% (*n* = 54)	
Retinopathy				
Yes	30.3% (*n* = 91)	34.3% (*n* = 37)	28.1% (*n* = 54)	0.267
No	69.7% (*n* = 209)	65.7% (*n* = 71)	71.9% (*n* = 138)	
Other co-morbidities				
Yes	76.3% (*n* = 229)	71.3% (*n* = 77)	79.2% (*n* = 152)	0.124
No	23.7% (*n* = 71)	28.7% (*n* = 31)	20.8% (*n* = 40)	

*Significant *p* values are in bold

### Analysis of OKT scores

The mean ± SD of OKT total score was 13.5 ± 4.2. The mean ± SD of exercise knowledge score was 8.8 ± 2.8 while that for nutrition knowledge score was 11 ± 3.6. Most of the participants correctly answered question 5 (OK5; 81.7%) and question 10 (OK10; 78%) (Table 2). Females had a significantly higher overall OKT score (*p* = 0.021), higher exercise knowledge score (*p* = 0.043), and higher nutrition knowledge score (*p* = 0.037) (Table 3).

**Table 2:** List of OKT questions with frequencies and percentage of patients giving the correct answer for each question

Variable*	Frequency of correctly answered (%)
OK1: Eating a diet low in dairy products and having osteoporosis تناول نظام غذائي يحوي كمية منخفضة من منتجات الالبان واصابتك بالهشاشه	170 (56.7%)
OK2: Being menopausal; “change of life” and having osteoporosis انقطاع الطمث” تغير في الحياة”واصابتك بالهشاشه	124 (41.3%)
OK3: Having a parent or grandparent who has osteoporosis and having osteoporosis ان يكون احد الوالدين او الجدين مصاب بهشاشة العظام واصابتك بالهشاشه	117 (39.0%)
OK4: Being a white or Asian woman and having osteoporosis ان تكون امراة بيضاء او اسيوية واصابتها بالهشاشه	35 (11.7%)
OK5: Being an elderly man and having osteoporosis ان يكون رجل مسن واصابته بالهشاشه	245 (81.7%)
OK6: Having ovaries surgically removed and having osteoporosis ان تكون المبايض تم ازالتها جراحيا واصابتها بالهشاشه	61 (20.3%)
OK7: Taking cortisone (steroids e.g. Prednisone) for long time and having osteoporosis اخذ الكورتيزون(ستيرويد مثل بريدنيزون) لوقت طويل والاصابه بالهشاشه	195 (65.0%)
OK8: Being overweight and having osteoporosis زيادة الوزن والاصابه بالهشاشه	1 (0.3%)
OK9: Having an eating disorder and having osteoporosis ان يكون عنده اضطرابات في الاكل واصابته بالهشاشه	193 (64.3%)
OK10: Consuming more than 2 alcoholic drinks per day and having osteoporosis يستهلك اكثر من اثنين من المشروبات الكحولية كل يوم واصابته بالهشاشه	234 (78.0%)
OK11: Smoking on a daily basis and having osteoporosis التدخين بشكل يومي والاصابه بالهشاشه	180 (60.0%)
OK12: To strengthen bones, it is recommended that a person exercise at a moderately intense level for 30 min a day at least لتقوية العظام, يوصى ان يقوم الشخص بممارسة الرياضة بشكل متوسط من الكثافة لمدة 30 دقيقة يوميا على الأقل	113 (37.7%)
OK13: Exercise makes bones strong, but it must be hard enough to make breathing ممارسة الرياضة تجعل العظام قوية, لكن يجب ان تكون الرياضة بجهد كاف لجعل التنفس	163 (54.3%)
OK14: Which of the following activities is the best way to reduce a person’s chance of getting osteoporosis أي من النشاطات التالية هي افضل طريقة لتقليل فرصة إصابة الشخص بهشاشة العظام	143 (47.7%)
Ok15: Which of the following activities is the best way to reduce a person’s chance of getting osteoporosis أي من النشاطات التالية هي افضل طريقة لتقليل إصابة الشخص بهشاشة العظام	28 (9.3%)
Ok16: Which of the following activities is the best way to reduce a person’s chance of getting osteoporosis أي من النشاطات التالية هي افضل طريقة لتقليل إصابة الشخص بهشاشة العظام	173 (57.7%)
OK17: Which of the following activities is the best way to reduce a person’s chance of getting osteoporosis أي من النشاطات التالية هي افضل طريقة لتقليل إصابة الشخص بهشاشة العظام	117 (39.0%)
Ok18: Which of these is the best source of calcium أي مما يلي هو افضل مصدر للكالسيوم	233/ (77.7%)
OK19: Which of these is the best source of calcium أي مما يلي هو افضل مصدر للكالسيوم	160 (53.3%)
OK20: Which of these is the best source of calcium أي مما يلي هو افضل مصدر للكالسيوم	167 (55.7%)
OK21: Which of these is the best source of calcium أي مما يلي هو افضل مصدر للكالسيوم	222 (74.0%)
OK22: Which of these is the best source of calcium أي مما يلي هو افضل مصدر للكالسيوم	77 (25.7%)
OK23: Which of the following is the recommended amount of calcium intake for an adult per day أي مما يلي هي الكمية الموصى بها من الكالسيوم للبالغين يوميا	21 (7.0%)
Ok24: How much milk must an adult drink to meet the recommended amount of calcium ما هي كمية الحليب التي يجب على الانسان البالغ تناولها لتلبية كمية الكالسيوم الموصى بها	48 (16.0%)
OK25: Which of the following is the best reason for taking a calcium supplement أي مما يلي هو افضل سبب لاخذ مكملات الكالسيوم	118 (39.3%)
OK26: Which vitamin is required for the absorption of calcium أي من الفيتامينات مطلوب للامتصاص الكالسيوم	113 (37.7%)
OK27: Which is the best source of the vitamin required for the absorption of calcium (carrots, oranges, sunlight, don’t know) ما هو افضل مصدر للفيتامين اللازم لامتصاص الكالسيوم	127 (2.3%)
OK28: Which is the best food source of the vitamin required for the absorption of calcium ما هو افضل مصدرغذائي للفيتامين اللازم لامتصاص الكالسيوم	78 (26.0%)
OK29: Which of the following is the recommended amount of the vitamin required for the absorption of calcium for an adult, 50 years old and older أي مما يلي هي الكمية الموصى بها من الفيتامين اللازم للامتصاص الكالسسيوم عند البالغين من العمر خمسين سنة او اكثر	17 (5.7%)
OK30: When is the best time to build strong bones ما هو افضل وقت لبناء عظام قويه	23 (7.7%)
OK31: Osteoporosis can be diagnosed by هشاشه العظام يتم تشخيصها من خلال	120 (40.0%)
OK32: once you have osteoporosis بمجرد الإصابة بهشاشة العظام	213 (71.0%)

OK(number) = number of the question in osteoporosis knowledge test

*Questions from 1 to 12 were statement related to likelihood to get osteoporosis and the participants need to answer with “more likely”, “less likely”, “neutral”, or “do not know”. Questions from 13 to 32 were multiple-choice questions and the participants need to pick the correct answer

**Table 3:** Scores of osteoporosis knowledge subscales examined by gender

Variable	(M ± SD or %) *N* = 300	Male; *N* = 108 (M ± SD or %)	Female; *N* = 192 (M ± SD or %)	P*
Knowledge Scores				
Total (M/SD)	13.5 ± 4.2	12.7 ± 4.0	13.9 ± 4.2	**0.021**
- Nutrition (M/SD)	11 ± 3.6	10.4 ± 3.6	11.3 ± 3.6	**0.037**
- Exercise (M/SD)	8.8 ± 2.8	8.4 ± 2.7	9.1 ± 2.8	**0.043**

*Significant *p* values are in bold

### Analysis of OHBS scores

The mean ± standard deviation for osteoporosis belief subscales are shown in Table 4. Diabetic patients had the highest score (mean ± SD = 23.1 ± 2.5) in belief subscale pertaining to benefit of exercise as protective against osteoporosis. On the other hand, diabetic patients had the lowest score in belief subscale pertaining to the presence of barriers to calcium intake. Examples of frequency of answers for certain questions in OHBS include the followings. (1) more than half (54%) of participants did not believe that they have high chances of getting osteoporosis (item #1); (2) the majority (78.4%) of participants incorrectly answered that family history makes them more likely to have osteoporosis (item # 6); (3) 46% agreed that it would be scary to get osteoporosis (item # 7); (4) 75% correctly answered that exercise is protective toward osteoporosis (item # 14); (5) 85% correctly answered that calcium intake is protective toward osteoporosis (item # 19); (6) 68% agreed that it would be serious to get osteoporosis (item # 12). The majority of participants disagreed with the five statements of perceived barriers to exercise (items number 26–30), but the majority agreed that their physical weakness is a perceived barrier to exercise (item # 25). Similarly, the majority of participants disagreed with the five statements of the perceived barriers to take calcium (items number 31–35). However, the majority agreed that food rich in calcium might be rich in cholesterol and therefore was a perceived barrier to intake of calcium rich food (item # 36). The majority of participants agreed to all statements that assess health motivation (items number 37–42).

**Table 4:** Osteoporosis belief scores examined by gender

Variable	(M ± SD or %) *N* = 300	Males; *N* = 108 (M ± SD or %)	Females; *N* = 192 (M ± SD or %)	P*
Belief Subscale				
Total belief construct	136.1 ± 10.0	133.9 ± 9.9	137.4 ± 9.8	**< 0.01**
- Susceptibility (M/SD)	16.9 ± 4.0	15.8 ± 3.8	17.4 ± 4.1	**< 0.01**
- Seriousness	19.4 ± 3.6	18.7 ± 3.6	19.8 ± 3.6	**< 0.01**
- Benefit of exercise	23.1 ± 2.5	23.0 ± 2.8	23.0 ± 2.3	0.78
- Benefit of calcium	22.8 ± 2.4	22.6 ± 2.5	23.0 ± 2.3	0.27
- Barrier to exercise	16.6 ± 3.5	16.3 ± 5.5	15.1 ± 30	0.18
- Barrier to calcium	15.4 ± 2.7	15.1 ± 2.9	15.5 ± 2.6	0.18
- Health motivation	22.0 ± 2.8	22.4 ± 2.9	21.8 ± 2.8	0.104

M ± SD = mean ± standard deviation

*Significant *p* values are in bold

When belief scores were examined by gender, female participants showed significantly higher scores in perceived susceptibility (17.4 ± 4.1 for females versus 15.8 ± 3.8 for males; *p* <  0.01) and perceived seriousness (19.8 ± 3.6 for females versus 18.7 ± 3.6 for males; *p* < 0.01) of osteoporosis when compared to male participants. No significant difference was found between males and females in other belief subscales (Table 4).

## Discussion

This study was carried out to assess osteoporosis knowledge and beliefs in diabetic patients in a governmental diabetic primary health care center in Nablus, Palestine. The levels of osteoporosis knowledge and perception were poor necessitating preventive educational programs to increase awareness about risks of osteoporosis. In particular, these preventive measures could target young diabetic females and those who are unemployed since they have poor perception toward osteoporosis.

Our results regarding poor knowledge and perception were expected given that the majority of participants were not well educated. Studies from non-Arab Middle Eastern countries such as Turkey and Iran highlighted that educational level of females can determine the extent of awareness of osteoporosis [45, 46]. Our results indicated that the majority of participants failed to answer correctly 19 questions of OKT suggesting that participants will not be able to get engaged in behaviors and practices that will decrease their risk of osteoporosis. Potential reasons for poor knowledge among participants were most probably poor educational level.

In our study, both males and females did not believe that they were susceptible to osteoporosis. Furthermore, participants did not have a high perception of seriousness of osteoporosis. The relatively poor perception toward susceptibility and seriousness of osteoporosis could be due, low awareness and/or poor health promotion system in Palestine. In Palestinian ministry of health had launched several awareness workshops and campaigns to fight several diseases such as breast cancer, smoking, childhood vaccination. However, osteoporosis awareness campaigns and workshops and research are absent, not only in Palestine but in Arab countries as well [47]. Our findings might necessitate implementing a periodic screening for bone mineral density in diabetic patients and implementing nurse or physician – patient communication about osteoporosis and potential serious consequences of bone fractures.

To the best of our knowledge, no previous studies about knowledge or belief about osteoporosis among diabetic patients had been published from Arab countries, and therefore, comparison of our results with published studies in other Arab communities was not possible. However, several studies have been published about knowledge and belief about osteoporosis among different groups of women or particular types of patients [29, 48–53]. A study on osteoporosis knowledge among Chinese HIV patients indicated that osteoporosis knowledge was universally low and that participants with lower education perceived greater barriers to implement preventive behaviors [30].

Our study has limitations and points of weaknesses. The cross-sectional design and convenience sampling technique used to recruit the participants could have created some bias. Future studies need to include larger sample of DM patients with various educational background and from different regions in Palestine. Furthermore, future studies need to shed light on daily practices of patients with regard to nutrition such as protein and calcium intake to link belief with daily practices.

## Conclusions

Overall, diabetic patients had poor osteoporosis knowledge, moderate perception of susceptibility and seriousness of osteoporosis despite that they have relative high score in health motivation subscale. Implementation of awareness and educational programs among diabetic patients might increase preventive practices and measures toward osteoporosis. Such preventive practices need to focus on calcium rich nutrition and regular exercise.

## Additional file

Additional file 1: Arabic translation of osteoporosis knowledge and beliefs tests. The file includes the questionnaire used in the survey study with the Arabic translation of the knowledge and belief tests. (DOCX 27 kb)

## Abbreviations

DM: Diabetes mellitus
IRB: Institutional Review Board
OHBS: Osteoporosis Health Belief Scale
OKT: Osteoporosis Knowledge Test
