# Supplementary material for: Osteoporosis knowledge and beliefs in diabetic patients: a cross sectional study from Palestine
Source: BMC Musculoskelet Disord. 2018 Feb 7;19:43. doi: 10.1186/s12891-018-1961-6 (PMC5803863; doi:10.1186/s12891-018-1961-6)
Supplement: Additional file 1: — Arabic translation of osteoporosis knowledge and beliefs tests. The file includes the questionnaire used in the survey study with the Arabic translation of the knowledge and belief tests. (DOCX 27 kb) [file 12891_2018_1961_MOESM1_ESM.docx]

Additional file 1: Osteoporosis knowledge and beliefs tests

استبيان حول معلومات شخصية وسريرية عن المشاركين في البحث

Demographic and clinical data of the participant

معلومات شخصية : Personal Information

الاسم (Name ) : .........................

العمر ( Age) : ..........................

الجنس (Gender ):..........................

مكان السكن (Place of living): ..................

طبيعة العمل (Occupation): ..................

الحالة الاجتماعية (Marital status): .............

- مدة الإصابة بمرض السكرى؟ Duration of having Diabetes Mellitus

أ_ 0- 5 سنين. 0 – 5 years

ب_ 6- 10 سنة . 6 – 10 years

ج_ 11≤ سنة . ≥ Years

- طبيعة الدواء المستخدم لمعالجة مرض السكري ؟ Medications used for diabetes mellitus

أ_ انسولين.Insulin

ب_ حبوب . Oral hypoglycemic agents

ج_ انسولين وحبوب . Both Insulin and oral hypoglycemic agents

- مؤشر كتلة الجسم (BMI)

أ_ نحيف (اقل من 18.5). < 18.5

ب_ طبيعي او صحي ( من 18.5 الى 24.9). 18.5 – 24.9

ج_ زيادة وزن ( من 25 الى 29.9). Overweight 25 – 29.9

د_ سمين ( اكثر من 30). Obese > 30

- هل تعاني من ارتفاع ضغط الدم ؟ Do you have hypertension أ_ نعم Yes ب_ لا No
- هل تعاني من امراض كلى ؟ Do you have kidney problems أ_ نعم Yes ب_ لا No
- هل تعاني من امراض في العين ؟ Do you have eye problems أ_ نعم Yes ب_ لا No

هل تعاني من امراض أخرى ؟ Do you suffer from other disease?

The following list of questions is the Arabic translation of the Osteoporosis Knowledge Test (OKT) as obtained from the developer.

Below is a list of things (questions 1 to 11) which may or may not affect a person's chance of getting osteoporosis. After you read each statement, think about if the person is:

MORE LIKELY TO GET OSTEOPOROSIS, or

LESS LIKELY TO GET OSTEOPOROSIS, or

NEUTRAL, IT HAS NOTHING TO DO WITH GETTING OSTEOPOROSIS, or

DON’T KNOW.

When you read each statement, circle ONE of the 4 choices for your answer.

هشاشة العظام هي عبارة عن حالة تصبح فيها العظام رقيقة بشكل مفرط وضعيفة بحيث تكون معرضة للكسر بسهولة .

فيما يلي قائمة بالاشياء التي يمكن ان تؤثر او لا تؤثر على فرصة الشخص بالاصابة بهشاشة العظام . بعد قراءة كل عبارة , فكر اذا كان الشخص : اكثر عرضة للإصابة بهشاشة العظام , او اقل عرضة للإصابة بهشاشة العظام , او محايد (ليس له علاقة بالاصابة بهشاشة العظام ) , او انك لا تعرف .

عندما تقرا كل عبارة ضع دائرة حول إجابة واحدة من الخيارات الأربعة

1. تناول نظام غذائي يحوي كمية منخفضة من منتجات الالبان ؟ Eating a diet LOW in dairy products

ا. اكثر عرضة more likely

ب. اقل عرضة less likely

ج. محايد neutral

د. لا اعرف do not know

2. انقطاع الطمث" تغير في الحياة"؟ Being menopausal; "change of life"

ا. اكثر عرضة more likely

ب. اقل عرضة less likely

ج. محايد neutral

د. لا اعرف do not know

3. ان يكون احد الوالدين او الجدين مصاب بهشاشة العظام ؟ Having a parent or grandparent who has osteoporosis

ا. اكثر عرضة more likely

ب. اقل عرضة less likely

ج. محايد neutral

د. لا اعرف do not know

4. ان تكون امراة بيضاء او اسيوية ؟ Being a white or Asian woman

ا. اكثر عرضة more likely

ب. اقل عرضة less likely

ج. محايد neutral

د. لا اعرف do not know

5. ان يكون رجل مسن ؟ Being an elderly man

ا. اكثر عرضة more likely

ب. اقل عرضة less likely

ج. محايد neutral

د. لا اعرف do not know

6. ان تكون المبايض تم ازالتها جراحيا ؟ Having ovaries surgically removed

ا. اكثر عرضة more likely

ب. اقل عرضة less likely

ج. محايد neutral

د. لا اعرف do not know

7. اخذ الكورتيزون( ستيرويد مثل بريدنيزون) لوقت طويل ؟ Taking cortisone (steroids e.g. Prednisone) for long time

ا. اكثر عرضة more likely

ب. اقل عرضة less likely

ج. محايد neutral

د. لا اعرف do not know

8. زيادة الوزن ؟ Being overweight

ا. اكثر عرضة more likely

ب. اقل عرضة less likely

ج. محايد neutral

د. لا اعرف do not know

9. ان يكون عنده اضطرابات في الاكل ؟ Having an eating disorder

ا. اكثر عرضة more likely

ب. اقل عرضة less likely

ج. محايد neutral

د. لا اعرف do not know

10. يستهلك اكثر من اثنين من المشروبات الكحولية كل يوم ؟ Consuming more than 2 alcoholic drinks per day

ا. اكثر عرضة more likely

ب. اقل عرضة less likely

ج. محايد neutral

د. لا اعرف do not know

11. التدخين بشكل يومي ؟ Smoking on a daily basis

ا. اكثر عرضة more likely

ب. اقل عرضة less likely

ج. محايد neutral

د. لا اعرف do not know

بالنسبة للمجموعة التالية من الأسئلة , ضع دائرة حول إجابة واحدة من الخيارات .تاكد ان تضع دائرة حول إجابة واحدة فقط . اذا كنت تعتقد ان هناك اكثر من إجابة واحدة صحيحة , اختار افضل إجابة . اذا لم تكن متأكد , ضع دائرة حول الرمز (د) لا اعرف .

For the next group of questions, circle one answer from the 4 choices. Be sure to circle ONLY ONE answer. If you think there is more than one correct answer, choose the BEST answer. If you are not sure, circle D. Don’t know.

12. لتقوية العظام , يوصى ان يقوم الشخص بممارسة الرياضة بشكل متوسط من الكثافة لمدة 30 دقيقة يوميا على الأقل ؟

To strengthen bones, it is recommended that a person exercise at a moderately intense level for 30 minutes a day at least

ا. 3 أيام في الأسبوع 3 days a week

ب. 4 أيام في الأسبوع 4 days a week

ج. 5 أيام في الأسبوع 5 days a week

د. لا اعرف do not know

13. ممارسة الرياضة تجعل العظام قوية , لكن يجب ان تكون الرياضة بجهد كاف لجعل التنفس ؟

Exercise makes bones strong, but it must be hard enough to make breathing

ا. اسرع بقليل فقط Just a little faster

ب. اسرع بكثير , لكن من الممكن ان يتحدث Much faster, but talking is possible

ج. سريع لدرجة ان الكلام غير ممكن So fast that talking is not possible

د. لا اعرف do not know

14. أي من النشاطات التالية هي افضل طريقة لتقليل فرصة إصابة الشخص بهشاشة العظام ؟

Which of the following activities is the best way to reduce a person’s chance of getting osteoporosis?

ا. السباحة Swimming

ب. المشي السريع Walking briskly

ج. التمدد Stretching

د. لا اعرف do not know

15. أي من النشاطات التالية هي افضل طريقة لتقليل إصابة الشخص بهشاشة العظام ؟

Which of the following activities is the best way to reduce a person's chance of getting osteoporosis?

ا. ركوب الدراجات Bicycling

ب. اليوغا ( نوع من تمارين اللياقة ) Yoga

ج. رفع الاثقال Lifting weights

د. لا اعرف do not know

16. أي من النشاطات التالية هي افضل طريقة لتقليل إصابة الشخص بهشاشة العظام ؟

Which of the following activities is the best way to reduce a person's chance of getting osteoporosis?

ا. هرولة او ركض Jogging or running

ب. لعب الغولف باستخدام عربة الغولف Golfing using golf cart

ج. زراعة الحدائق Gardening

د. لا اعرف do not know

17. أي من النشاطات التالية هي افضل طريقة لتقليل إصابة الشخص بهشاشة العظام ؟

Which of the following activities is the best way to reduce a person's chance of getting osteoporosis?

ا. البولينج( التصويب باتجاه الكرات باستخدام كرة أخرى ) Bowling

ب. غسل الملابس Doing laundry

ج. الرقص الهوائي Aerobic dancing

د. لا اعرف do not know

18. أي مما يلي هو افضل مصدر للكالسيوم ؟

Which of these is the best source of calcium?

ا. التفاح Apple

ب. الجبنة Cheese

ج. الخيار Cucumber

د. لا اعرف do not know

19. أي مما يلي هو افضل مصدر للكالسيوم ؟

Which of these is the best source of calcium?

ا. زبدة الفول السوداني Peanut Butter

ب. الديك الرومي Turkey

ج. السردين المعلب Canned Sardines

د. لا اعرف do not know

20. أي مما يلي هو افضل مصدر للكالسيوم ؟

Which of these is the best source of calcium?

ا. الدجاج Chicken

ب. البروكلي Broccoli

ج. العنب Grapes

د. لا اعرف do not know

21. أي مما يلي هو افضل مصدر للكالسيوم ؟

Which of these is the best source of calcium?

ا. لبن الزبادي Yogurt

ب. فراولة Strawberries

ج. كرنبيط ( ملفوف) Cabbage

د. لا اعرف do not know

22. أي مما يلي هو افضل مصدر للكالسيوم ؟

Which of these is the best source of calcium?

ا. البوظة (ايس كريم) Ice cream

ب. فاكهة العنب Grape fruit

ج. الفجل Radishes

د. لا اعرف do not know

23. أي مما يلي هي الكمية الموصى بها من الكالسيوم للبالغين ؟

Which of the following is the recommended amount of calcium intake for an adult?

ا. 600-800 ملغ يوميا 600 mg – 800 mg daily

ب. 1000-1200 ملغ يوميا 1000 mg – 1200 mg daily

ج. 1400-1600 ملغ يوميا 1400 mg – 1600 mg daily

د. لا اعرف do not know

24. ما هي كمية الحليب التي يجب على الانسان البالغ تناولها لتلبية كمية الكالسيوم الموصى بها ؟

How much milk must an adult drink to meet the recommended amount of calcium?

ا. كوب واحد يوميا one glass daily

ب. كوبان يوميا two glasses daily

ج. ثلاثة اكواب او اكثر three or more glasses daily

د. لا اعرف do not know

25. أي مما يلي هو افضل سبب لاخذ مكملات الكالسيوم ؟

Which of the following is the best reason for taking a calcium supplement?

ا. اذا كان الشخص لا يتناول وجبة الفطور If a person skips breakfast

ب. اذا كان الشخص لا يحصل على كمية كافية من الكالسيوم من الغذاء If a person does not get enough calcium from diet

ج. اذا كان الشخص فوق الأربعين سنة If a person is over 45 years old

د. لا اعرف do not know

26. أي من الفيتامينات مطلوب للامتصاص الكالسيوم ؟

Which vitamin is required for the absorption of calcium?

ا. فيتامين أ Vitamin A

ب. فيتامين ج Vitamin C

ج. فيتامين د Vitamin D

د. لا اعرف do not know

27. ما هو افضل مصدر للفيتامين اللازم للامتصاص الكالسيوم ؟

Which is the best source of the vitamin required for the absorption of calcium?

ا. الجزر Carrots

ب. البرتقال Oranges

ج. ضوء الشمس Sunlight

د. لا اعلم do not know

28. ما هو افضل مصدر غذائي للفيتامين اللازم للامتصاص الكالسيوم ؟

Which is the best food source of the vitamin required for the absorption of calcium?

ا. السبانخ Spinach

ب. الجبنة Cheese

ج. سمك السلمون Salmon

د. لا اعرف do not know

29. أي مما يلي هي الكمية الموصى بها من الفيتامين اللازم للامتصاص الكالسسيوم عند البالغين من العمر خمسين سنة او اكثر ؟

Which of the following is the recommended amount of the vitamin required for the absorption of calcium for an adult, 50 years old and older?

ا. 800-1000 وحدة دولية يوميا 800 – 1000 IU daily

ب. 1200-1400 وحدة دولية يوميا 1200 – 1400 IU daily

ج. 1600-1800 وحدة دولية يوميا 1600 – 1800 IU daily

د. لا اعرف do not know

30. ما هو افضل وقت لبناء عظام قوية ؟

When is the best time to build strong bones?

ا. مرحلة الطفولة Childhood

ب. مرحلة المراهقة Adolescence

ج. مرخلة البلوغ Young adulthood

د. لا اعرف do not know

31. هشاشة العظام يمكن تشخيصها من خلال ؟

Osteoporosis can be diagnosed by

ا. فحص الدم Blood test

ب. المسح الضوئي لكثافة العظام DXA test

ج. الاعراض Symptoms

د. لا اعرف do not know

32. بمجرد الإصابة بهشاشة العظام ؟

Once you have osteoporosis

ا. لا يوجد شيْ يمكنك القيام به تجاة ذلك There is nothing you can do about it

ب. يمكنك ان تأخذ دواء لعلاجه You can take medication to treat it

ج. يجب ان تكون حذرا من رفع الأشياء You must be careful lifting objects

د. لا اعلم do not know

**OSTEOPOROSIS HEALTH BELIEF SCALE**

For each of the following list of sentences please indicate your opinion as: Strongly disagree, Disagree, Neutral, Agree, Strongly agree.

لكل سؤال من الإسئله التاليه، عبر عن رأيك كالتالي: لا أوافق بشده، لا أوافق، محايد، أوافق، أوافق بشده

| فرصتك في الإصابة بهشاشة العظام عالية ؟  chances of getting osteoporosis high |
| --- |
| بسبب بنية جسمك فانك اكثر عرضة للإصابة بهشاشة العظام؟  body build likely to get osteoporosis |
| من المحتمل جدا ان تصاب بهشاشة عظام ؟  extremely likely to get osteoporosis |
| هناك فرصة جيدة لك للإصابة بهشاشة العظام ؟  good chance will get osteoporosis |
| انت اكثر عرضة من الشخص العادي للإصابة بهشاشة العظام؟  more likely than average person to get osteoporosis |
| تاريخ عائلتك يجعلك اكثر عرضة للإصابة بهشاشة العظام؟  family history makes it more likely will get osteoporosis |
| التفكير بالاصابة بهشاشة العظام يخيفك؟  thought of having osteoporosis scares me |
| اذا كان لديك هشاشة عظام يمكن ان تصاب بشلل؟  if had osteoporosis would be crippled |
| مشاعرك تجاه نفسك سوف تتغير اذا اصبت بهشاشة العظام؟  feeling about self would change if got osteoporosis |
| سوف يكون مكلفا للغاية اذا اصبت بهشاشة العظام؟  would be costly if got osteoporosis |
| عندما تفكر بهشاشة العظام تصاب بالاكتئاب؟  when think about osteoporosis, get depressed |
| سوف يكون خطير للغاية اذا اصبت بهشاشة العظام؟  would be serious if got osteoporosis |
| ممارسة التمارين الرياضية بشكل منتظم يمنع المشاكل التي قد تحدث من هشاشة العظام؟  regular exercise prevents problems of osteoporosis |
| تشعر بانك افضل عند ممارسة الرياضة لمنع هشهاشة العظام؟  feel better when exercise to prevent osteoporosis |
| ممارسة الرياضة بشكل منتظم يساعد على بناء عظام قوية؟  regular exercise helps build strong bones |
| ممارسة الرياضة لمنع هشاشة العظام يحسن من الطريقة التي يبدو بها جسمك؟  exercise for osteoporosis also improves body looks |
| ممارسة الرياضة بشكل منتظم يقلل من فرص كسر العظام؟  regular exercise cuts down chances of broken bones |
| تشعر بالرضا عن نفسك عندما تمارس الرياضة لمنع هشاشة العظام؟  feel good about self when exercise to prevent osteoporosis |
| اخذ ما يكفي من الكالسوم يمنع مشاكل هشاشة العظام؟  taking enough calcium prevents problems of osteoporosis |
| لديك الكثير لتكسبه من تناول ما يكفي من الكالسيوم لمنع هشاشة العظام؟  have lots to gain taking enough calcium to prevent osteo |
| اخذ ما يكفي من الكالسيوم يمنع هشاشة العظام المؤلمة؟  taking enough calcium prevents painful osteoporosis |
| لن تقلق كثيرا حول هشاشة العظام اذا اخذت ما يكفي من الكالسيوم؟  wouldn't worry as much about osteoporosis if enough calcium |
| اخذ ما يكفي من الكالسيوم يقلل من فرصك للإصابة بكسور العظام؟  taking enough calcium cuts chance of broken bones |
| تشعر بالرضا عن نفسك عندما تأخذ ما يكفي من الكالسيوم لمنع هشاشة العظام؟  feel good about self when enough calcium to prevent osteoporosis |
| تشعر بانك لست قوي ما يكفي لممارسة الرياضة بشكل منتظم؟  feel like not strong enough to exercise regularly |
| لا يوجد لديك مكان تستطيع ممارسة الرياضة فيه؟  have no place where can exercise |
| زوجك او عائلتك لا يشجعوك على ممارسة الرياضة؟  spouse/family discourage from exercising |
| ممارسة الرياضة بانتظام يعني البدء بعادة جديدة من الصعب عليك ان تفعلها؟  exercising regularly is new habit - hard to do |
| ممارسة الرياضة بشكل منتظم يجعلك غير مستريح؟  exercising regularly makes uncomfortable |
| ممارسة الرياضة بشكل منتظم يخل من الروتين اليومي الخاص بك ؟  exercising regularly upsets everyday routine |
| لأطعمة الغنية بالكالسيوم تكلف كثيرا ؟  calcium rich foods cost too much |
| الأطعمة الغنية بالكالسيوم لا توافقني؟  calcium rich food don’t agree with me |
| لا تحب الأطعمة الغنية بالكالسيوم؟  do not like calcium rich foods |
| تناول الأطعمة الغنية بالكالسيوم يعني تغيير النظام الغذائي الخاص بك وهذا من الصعب القيام به؟  eating calcium rich foods diet change-hard to do |
| من اجل تناول المزيد من الأطعمة الغنية بالكالسيوم عليك ان تتخلى عن اطعمة أخرى تحبها؟  to eat more calcium foods have to give up other I like |
| الأطعمة الغنية بالكالسيوم تحتوي على الكثير من الكولسترول؟  calcium rich foods have too much cholesterol |
| تاكل نظام غذائي متوازن؟  eat well balanced diet |
| انت تبحث عن معلومات جديدة متعلقة بالصحة؟  look for new information related to health |
| البقاء بصحة جيدة مهم جدا بالنسبة لك؟  keeping health is very important for me |
| تحاول ان تكتشف المشاكل الصحية في وقت مبكر؟  try to discover health problems early |
| لديك فحوصات طبية بشكل منتظم حتى وان لم تكن مريضا؟  have regular health check-up even when not sick |
| انت تتبع التوصيات من اجل ان تبقى بصحة جيدة؟  follow recommendations to keep me healthy |
